# Phantom-based training of ultrasound-guided breast biopsy in medical education: a randomized controlled trial comparing handheld and high-end ultrasound

**DOI:** 10.1186/s12909-025-07163-1

**Published:** 2025-04-16

**Authors:** Barbara Greiner, Michael Akers, Florian Zeman, Andrea Goetz, Moritz Brandenstein, Christian Stroszczynski, Ernst Michael Jung, Simone Hammer

**Affiliations:** 1 Department of Radiology, University Hospital Regensburg, Regensburg, Germany; 2 Department of Radiology, University Hospital Erlangen, Regensburg, Germany; 3 Center for Clinical Studies, University Medical Center Regensburg, Regensburg, Germany

## Abstract

**Background:**

Modern handheld ultrasound devices (HUDs) are attractive for teaching programs in undergraduate medical education due to their miniaturization and portability along at relatively low cost. They offer high-resolution imaging and are easy to use, opening up new possibilities for training of novices in ultrasound (US)-guided percutaneous procedures. The objective of this study was to investigate if guidance by HUD is on par with a cart-based high-end ultrasound scanner (HEUS) regarding performance times and success rates in video- and phantom-based training of novices in US-guided freehand breast biopsy.

**Methods:**

32 medical students without any experience in performing US-guided percutaneous biopsies, who had previously completed a standardized diagnostic US training program, were randomized into either a HUD-group (*n* = 16) or a HEUS-group (*n* = 16). After a video training lecture participants performed US-guided biopsies of hypoechogenic and hyperechogenic target-lesions in a breast phantom using either a HUD or a HEUS. Performance times and success rates were primary outcomes. Participants were asked to complete a post-study questionnaire (Likert Scale and Raw NASA Workload Task Load Index) for subjective assessment of the operability and individually perceived workload of both US imaging tools and guidance-techniques as secondary outcomes.

**Results:**

Biopsy success rates were slightly higher using the HUD (79.7%) in comparison to the HEUS (68.8%, *p* = 0.045). Median performance times were similar for the HUD (0.63 min, interquartile range IQR = 0.37–1.08 min) compared to the HEUS (0.60 min, IQR = 0.30–2.09 min, *p* = 0.751). Operability and the individually perceived workload were rated equal.

**Conclusions:**

Percutaneous biopsy performed by novices using HUDs is feasible, performance times, success rates, operability and the individually perceived workload were on par with HEUS-guidance. HUDs can be used as cost-effective tools for percutaneous biopsy training purposes in medical education.

**Supplementary Information:**

The online version contains supplementary material available at 10.1186/s12909-025-07163-1.

## Introduction

Major advances in US technology have enabled the widespread use of handheld ultrasound devices (HUDs) for many applications in various fields of clinical medicine [1, 2]. There is expanding literature describing the use of HUDs for core Point-of-Care-Ultrasound (POCUS) applications, including eFAST (extended focused assessment with sonography for trauma), identification of first trimester intrauterine pregnancy and other gynecological/obstetric indications, focused cardiac and thoracic US as well as procedural guidance of vascular access [1, 3, 4]. The probes can be carried in a lab coat and WLAN-connected to smartphones and tablets. Besides their lightweight nature and flexibility, HUDs are characterized by ease of handling, making them attractive for medical education training programs. As the use of bedside POCUS as extension of physical examination becomes customary, gaining familiarity with sonographic imaging techniques in medical school is considered to be beneficial [5]. Peer-to-peer programs for undergraduate POCUS training have been established at many institutions and scientifically evaluated [6]. However, current data still suggest a lack of structured undergraduate training in European medical universities, which is why the European Federation for Ultrasound in Medicine and Biology (EFSUMB) continues to promote the introduction of US as an integrative part of the core curriculum of medical education [7].

The various HUDs available on the market show specific characteristics such as the provided probes and software, e.g. Lumify (Philips Healthcare, Netherlands) has been rated with the highest image quality and Vscan Air (General Electric Healthcare, USA) the best in terms of ease of use [8]. In a recent comparison of eight different brands of HUDs, Vscan Air scored highest both in terms of B-Mode image quality and in terms of clinical significance [9]. Despite the rapid increase in usability and image resolution, imaging based on HUDs is still a complex and operator-dependent modality requiring standardized training und supervised practicing. Scientific data regarding the use of handheld US training involving undergraduate students are limited and heterogeneous. It is unclear whether HUDs are suitable for the training of novices [10]. Moreover, data on undergraduate training in US-guided percutaneous biopsy procedures are limited [11–13], and to the best of our knowledge, studies on biopsy training involving HUDs are lacking. To what extent and in what way HUDs can be integrated into medical undergraduates’ education still need to be clarified. While structured teaching in diagnostic US imaging as well as peer-to-peer programs have been running for years at our university hospital, no standardized practical interventional US training has been offered to medical students until now. This study aimed at evaluating if performance and success rates of novices conducting US-guided, video- and phantom-based breast biopsies differ depending on the used image guidance device (HUD vs. HEUS).

## Materials and methods

### Study design and participants

**Fig. 1:**
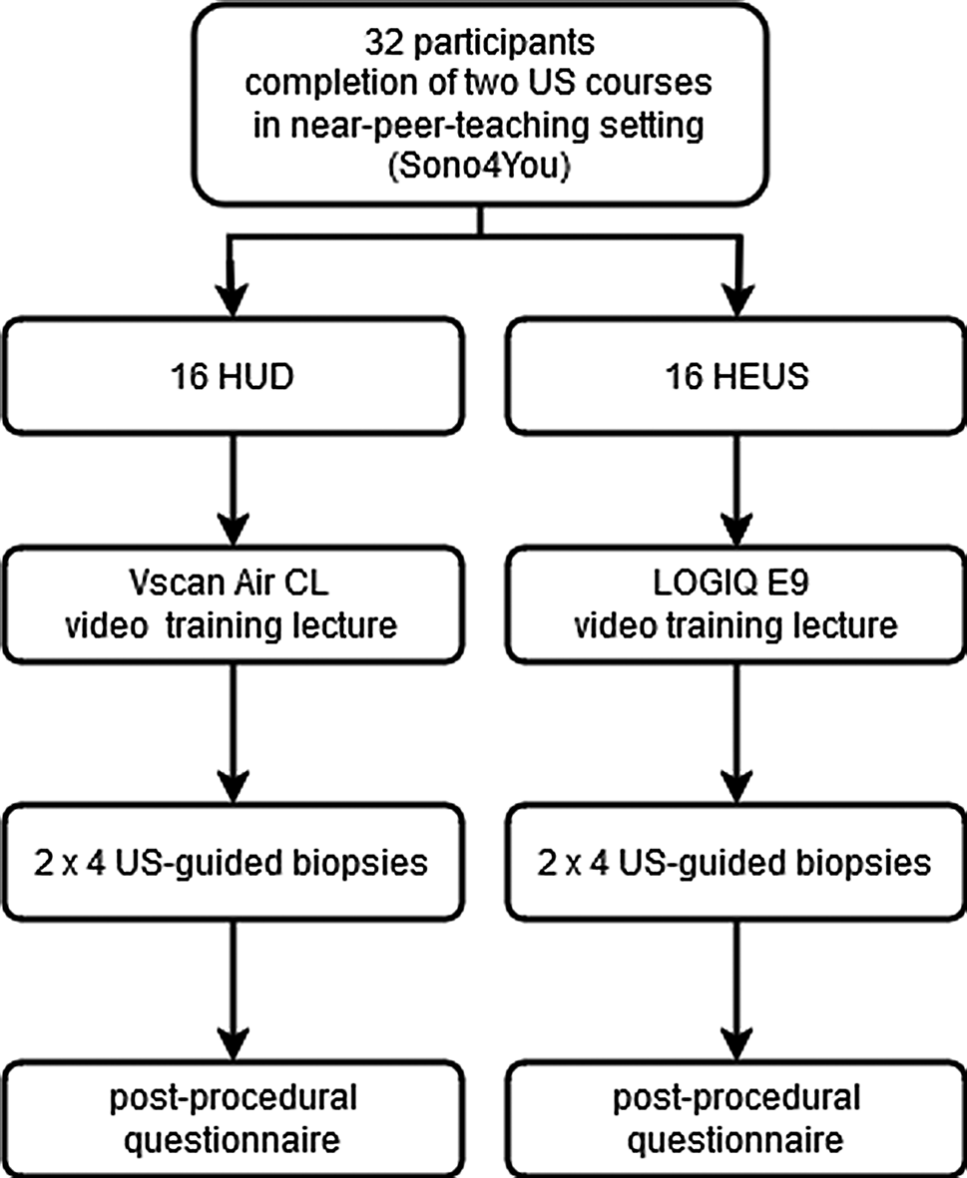
Diagrammatic representation of the study design

This single-center prospective randomized study was conducted at the Department of Radiology at the University Medical Center Regensburg between April 2024 and August 2024 after institutional review board approval. As this educational study did not involve clinical interventions it was not prospectively registered (Clinical trial number: not applicable). Medical Center teaching staff of the School of Medicine invited 120 medical students who had passed the first state examination and had attended a standardized training in diagnostic US. Obligatory for inclusion was the completion of two consecutive ultrasound courses in a near-peer-teaching setting, addressing basical as well as advanced knowledge of performing standardized ultrasound examinations on various body parts, though not covering interventional US-techniques so far [14, 15]. 32 medical students participated as volunteers in the study and written informed consent was obtained before study inclusion. Participants were randomly assigned to one of the two groups, the HUD-group or HEUS-group, using an envelope randomization strategy (Fig. 1).

The time slots, for which the participants were scheduled, were assigned to the HUD-group or HEUS-group by drawing envelope-concealed numbered assignment slips from an opaque pouch.

**Fig. 2:**
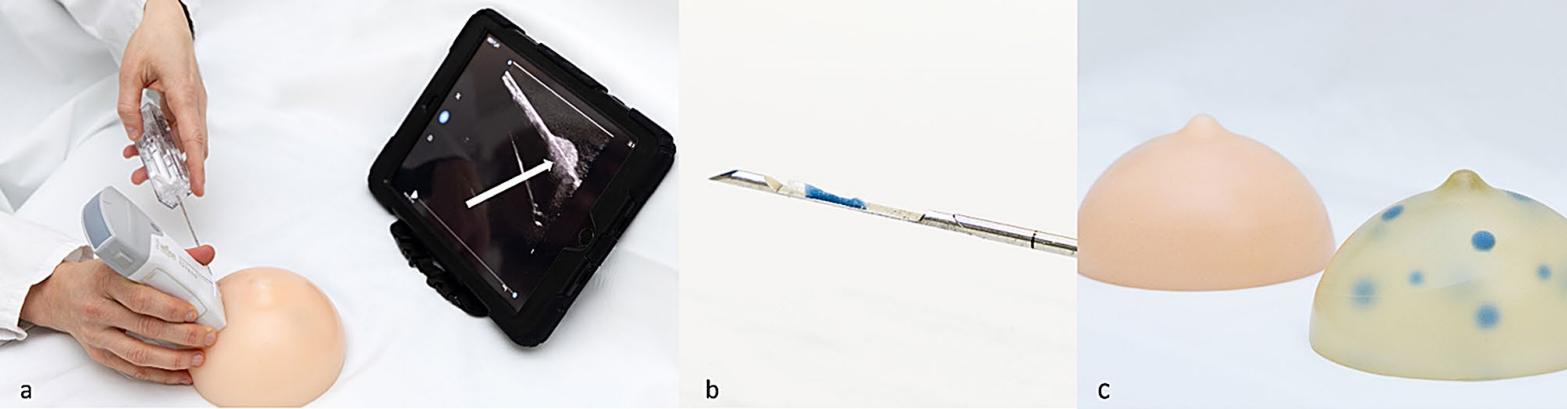
Successful biopsy. Post-fire image using handheld ultrasound guidance in **a**. Needle depicted in hyperechogenic lesion (arrow). Core sample with blue colored material indicating successful biopsy in **b**. Opaque (left and transparent (right) breast phantom in **c**

At the scheduled time slot the participants viewed one of two prerecorded training videos (approx. 9 min) providing a general overview of how US-guided interventions are performed and showing the specific task the participants were to perform (video #1 for using LOGIQ E9, and video #2 for using Vscan Air CL; both US-devices by General Electric Healthcare, Chicago, USA). The structure of the breast phantom and the target lesions were presented in the video. For standardization purposes participants received training solely through video presentation. Each participant watched the assigned video alone in a room, pausing or repeating it in whole or in part was not allowed. They could familiarize themselves with the assigned US-devices, using the linear probe of LOGIQ E9 with a frequency range of 9-14 MHz or Vscan Air with a frequency range of 3–12 MHz, the transparent and opaque breast phantoms (Kyoto Kagaku, Kyoto, Japan; Fig. 2c) and the biopsy needle (Achieve 14G, 11.0 cm long, Merit Medical, Limburg, Germany) during the video lecture and up to 5 min afterwards. The HUD was connected via WLAN to an ipad (Apple Inc., Cupertino, USA) solely serving for this purpose (Fig. 2a).

**Fig. 3:**
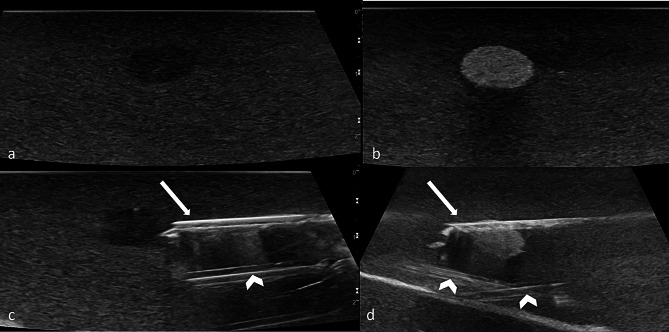
Target lesions. Hypoechogenic lesions in **a** and **c**, hyperechogenic lesions in **b** and **d**. Biopsy needle (arrows) pre-fire in c, post-fire in d. Hyperechogenic channels from previous biopsies (arrowheads)

Afterwards the participant scanned the breast phantom in B-Mode for identification of 4 target lesions: One 10 mm hypoechogenic and one 10 mm hyperechogenic lesion in the nipple-near part of the phantom, one 6 mm hyperechogenic lesion in the middle and one 10 mm hyperechogenic lesion in the deeper part of the phantom (Fig. 3). All participants had to biopsy the same 4 lesions in the same order starting with the 2 more superficial lying lesions and ending with the lesion in the deeper part of the phantom.

Once a target lesion was identified and adjusted, the preloaded biopsy needle was freehand-inserted into the phantom by the participant under US-guidance either using the HUD or the HEUS. At this timepoint (contact of the needle tip with the surface of the phantom) a timer was started by a supervisor, who was present in the same room without interacting with the participant. Time measurement was finished as soon as a core biopsy was obtained by firing the biopsy needle (Fig. 2a). A biopsy was deemed successful if any colored lesion material was present in the core sample (Fig. 2b). Four phantoms were used for the study (2 in each group) and the phantoms were exchanged for a new one in the middle of the study after the eighth participant in both groups. It had been outlined in the video training how to differentiate between tracks by prior biopsies and the inserted biopsy needle.

The procedure was repeated two times for each of the four lesions (in total 8 times per participant). Following the biopsy procedures, all participants were asked to complete a post-procedural questionnaire (Supplemental Fig. 1). For evaluating and assessing the participants` subjective impressions in the trial they had to record their personal experience in three categories. The Raw NASA Workload Task Load Index (TLX), a subjective, multidimensional assessment tool was used to rate the participants` perceived workload [16, 17]. The questionnaire`s design was chosen to be simple to facilitate the students´ assessment and minimize interobserver variability between the evaluators. Moreover, the standardized protocol including video-based training with as little interaction between observers and participants as possible and the choice of objective and simple parameters as primary outcomes were important factors addressing interobserver variability. For these reasons, the interobserver variability was not quantified.

### Primary outcomes

Primary outcomes of the study were the rate of positive biopsies per student and the skin-to-fire-time for each lesion.

### Secondary outcomes

The subjective assessment of the operability and safety of US imaging and biopsy guidance devices and individually perceived workload were defined as secondary outcomes. Directly after the biopsies were taken, the participants had to record their personal impressions in three categories with a 6-point Likert scale (0–5; 0 = absolutely comfortable/safe and not difficult at all, 5 = not at all comfortable resp. safe and very difficult): (1) Comfort (“I felt the imaging system used was comfortable for me”), (2) Safety (“I felt safe performing the biopsy while using this ultrasound imaging system“, regarding orientation, needle firing and tissue asservation), (3) Difficulty (“Overall difficulty of the task“). The assessment of the mental workload was performed with the Raw NASA Task Load Index (NASA TLX). Mental, physical and temporal demand, performance, effort and frustration of the participants were assessed on a 20-point Likert scale (1–20; 1 = very low, 20 = very high).

### Statistical analysis

Frequencies are presented as absolute numbers and percentages. Continuous data are presented as median values and interquartile ranges (IQRs). Differences between the groups were analyzed using the Pearson chi-square test of independence for dichotomous parameters and the Wilcoxon-Mann-Whitney U test for continuous data. A p-value < 0.05 was considered statistically significant. All analyses were performed using SPSS version 29.0.1.0 (IBM, NY, USA).

## Results

**Fig. 4:**
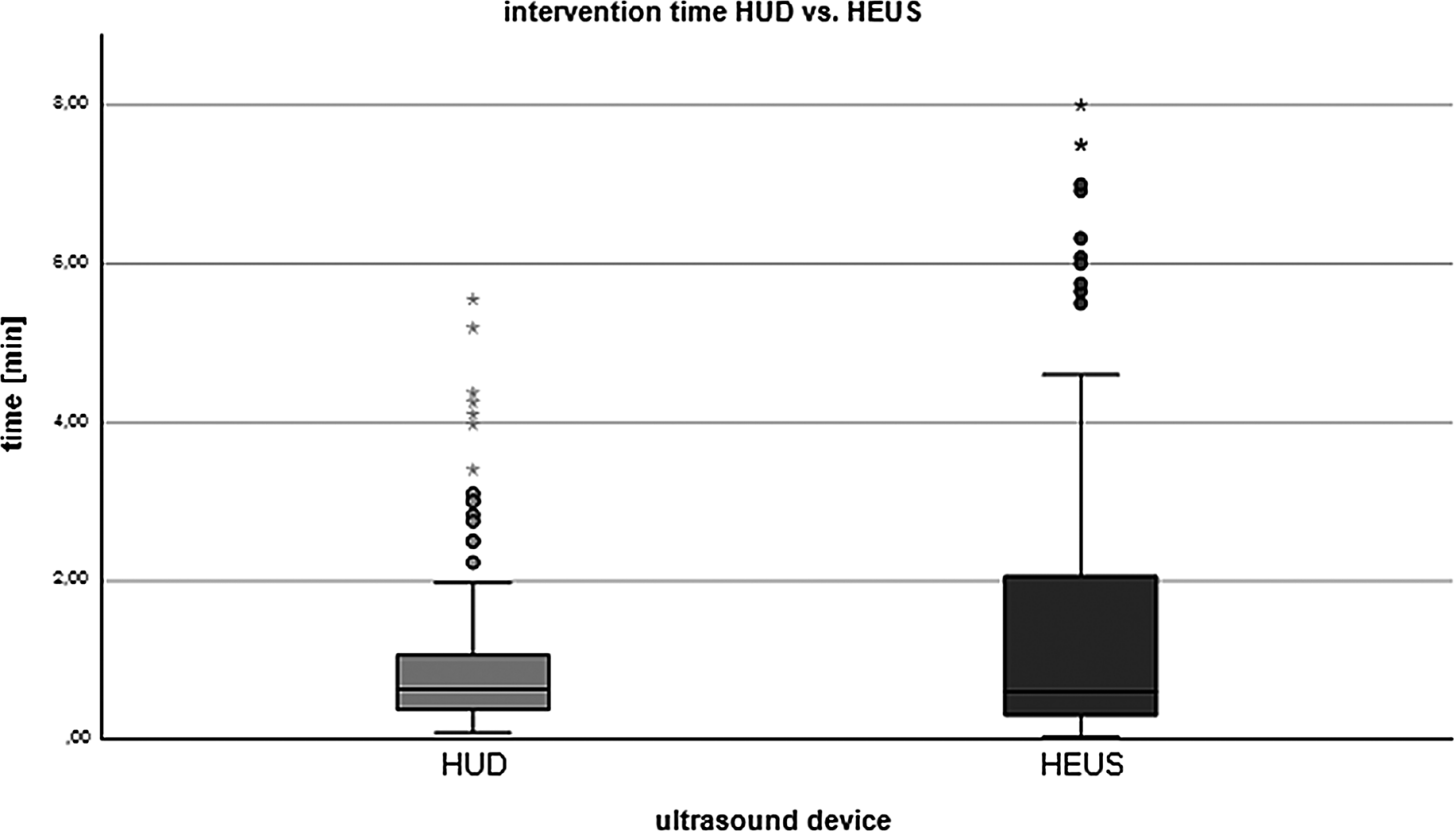
Boxplots showing intervention times for the devices used

Overall biopsy times were similar overall and for every single target lesion in the HUD-group (0.63 min, IQR = 0.37–1.08 min) and the HEUS-group (0.60 min, IQR = 0.30–2.09 min) with no statistically significant difference between the two devices (*p* = 0.751) (Fig. 4). For both devices median biopsy times for the last deep lying lesion were slightly longer than for the first superficial lying lesion: 0.70 min, IQR 0.44–1.71 (HUS) and 0.94 min, IQR 0.63–1.22 min (HUD) versus 0.54 min, IQR = 0.24–2.17 (HUS) and 0.77 min, IQR 0.44–1.19 min (HUD). Biopsies guided with the HUD were significantly more often successful in comparison to guidance with HEUS (79.9% versus 68.8%, *p* = 0.045).

With regard to the secondary outcomes comfort and safety, the HUD achieved slightly better ratings with no significant difference. The evaluation of mental workload assessment showed no significant differences between the ratings given by the two groups concerning all items (Table 1).

**Table 1:** Subjective assessment of the impressions regarding comfort, safety and difficulty and personal workload

	HUD-group (*n* = 16)	HEUS-group (*n* = 16)	*p*-value
**Personal impressions**			
Comfort	0.00 (0.00–1.00)	0.00 (0.00–1.00)	0.985
Safety	1.00 (0.25-2.00)	1.00 (0.25-3.00)	0.590
Difficulty	4.00 (2.00–3.00)	2.50 (2.00-3.75)	0.838
**Raw NASA Task Load Index**			
Mental demand	12.00 (8.00–14.00)	13.50 (9.00–15.00)	0.102
Physical demand	4.00 (3.00-9.25)	6.00 (4.25–11.50)	0.224
Temporal demand	6.00 (3.25-10.00)	7.00 (3.00–10.00)	0.926
Performance	7.00 (3.25–12.75)	7.00 (4.00-14-00)	0.867
Effort	12.50 (10.00–14.00)	12.50 (8.50-14.75)	0.838
Frustration	8.50 (2.50-13.75)	7.50 (3.00-10.75)	0.669

Data are presented as median with interquartile ranges. Personal impressions: 0 = absolutely comfortable/absolutely safe and not difficult at all, 5 = not at all comfortable/not at all safe and very difficult. Task load index: 1 = very low, 20 = very high

## Discussion

The feasibility of US-guided procedures carried out by medical students has been examined mostly with US-guided vascular access, much rarer with percutaneous biopsies [18]. Limited resources on staff and budget are opposed to a growing need for US teaching facilities, as pointed out by Schmidt et al., who assessed the effectiveness of a US breast training program including percutaneous biopsies on knowledge and skills of undergraduate medical students, concluding that medical education can be enhanced by teaching breast US skills [19].

The purpose of our initial feasibility study focused on the applicability of HUDs in novices regarding US-guided procedures and a more resource-saving setting such as training by video lectures. Our results suggest a non-inferiority of the used HUD compared to the HEUS for US-guided percutaneous breast phantom biopsy training. Biopsies guided with the HUD were slightly more successful than performed with the HEUS. We assume this small difference is due to technical advantages of the HUD such as probe flexibility or the individually adjustable positioning options of the used tablet, but random variability cannot be completely excluded due to the small study cohort. Certainly, a follow-up cross over-study with focus on the technical differences between the devices and including more participants would be beneficial in addressing this question.

No significant difference between the devices was found regarding overall biopsy times and the subjective assessment of comfort and safety in procedure guidance. Even though older data described a higher acceptance of HEUS amongst students compared to HUD for POCUS training [20], modern HUDs are used more frequently nowadays [21]. In our experimental setting the medical students who had passed their standardized diagnostic US training using HEUS rated their first experiences with HUD similar regarding the individually perceived workload, comfort and safety indicating a high acceptance for this new technique.

The question arises whether the use of HUDs could support peer-to-peer or near-peer education for undergraduate student training. The utilization of near-peers in the practice of ultrasound-guided procedures had been described as effective to develop sonography skills while reinforcing anatomy [22]. Medical education is one of the most interesting areas of application for HUD and has been described as a driving force for innovation of mobile US technique [4, 23]. Besides the ease of handling, cost-effectiveness is a major advantage compared to the usually more expensive cart-based systems, making it an attractive tool for students. HUDs have the potential to increase the availability of US training in medical education and to facilitate early exposure to US-guided procedure skills training by replacing the more expensive standard HEUS. Insufficient teaching on interventional radiology techniques in medical schools has been described as one reason for the workforce shortages in interventional radiology [24] and standardized curricula with a greater focus on interventional radiology have been suggested [25]. Lower costs of HUDs could ensure higher availability of devices used for hands-on interventional skills training. Given their ease of handling, additional training on the usage of HUDs specifically would probably not be required, but further research on the influence of HUDs on US training is needed. Future research should also focus on developing objective assessment criteria to measure the proficiency of ultrasound skills as stated by Recker et al. [26]. The long-term impacts of undergraduate handheld US training should be evaluated and evidence-based guidelines for targeted education need to be implemented. Methodological requirements for teaching studies have been recommended in order to establish future guidelines on US training in medical education [27].

Moreover, the comparatively low costs enable the use of portable devices in low-to middle income countries or other resource-limited settings, opening up new possibilities not only for general POCUS applications, but also for US-guided procedure training of undergraduate and graduate students [28–30]. Innovative low-cost solutions such as HUDs offer a safe, simple and sustainable solution toward building capacity for cancer control in low- to middle-income countries that at the moment mostly rely on blind or surgical biopsies for cancer diagnosis, as pointed out by Hricak et al. [31].

There are growing data on the use of deep learning algorithms to facilitate POCUS operations in resource-constrained settings [32]. Artificial intelligence may provide real-time coaching of image generation and interpretation in the future. In general, widespread use of artificial intelligence is expected to enhance the feasibility of prospective POCUS applications.

A prerequisite for the unrestricted use of HUDs in clinical routine is a secure image documentation. According to our experience, data transfer to and storage in the picture archiving and communication system as the traditional solution for image archiving are central issues that have not yet been solved satisfactorily. Cloud-based solutions for storage of de-identified data might be beneficial, especially in out-of-hospital situations, but still require regulatory challenges. While data security should be considered at all times when patients are involved, this topic can advantageously be neglected in the context of phantom- or simulator-based student training. In the setting of US skills training no requirements and legal regulations exist regarding image archiving. 

The phantoms used for this study reproduced the resistance and softness of breast tissue to a satisfactory degree. A minor limitation was the presence of hyperechogenic biopsy channels, which made depicting the needle tip during the biopsy slightly more difficult. However, this limitation was consistent across both groups, as two phantoms were used per group and were replaced with new ones midway through the study, after the eighth participant in each group.

A problem we had to face was that the HUD`s scanning time is limited by the battery [33]. At least two HUDs were needed to carry out the study, for one device usually had to be charged on site. This should be taken into account especially when several trainees are participating. It is to be expected that the restriction of scanning times can be reduced by higher battery capacities in the future. The limited number of participants has to be stated as a major limitation. This is due to the strict inclusion criteria which were defined to ensure the formation of two highly homogeneous study groups with the same experience level regarding US.

## Conclusions

We believe interventional US training should be part of the curriculum in medical education. Costs incurred could be significantly reduced and availability increased through the use of HUDs instead of cart-based machines. While this study showed a non-inferiority of HUDs regarding biopsy success rates, intervention times and the individually perceived comfort, safety and workload of the medical students in phantom-based percutaneous biopsy training, further research is needed on long-term skill-retention, standardized training protocols and the value of AI-assisted guidance.

## Electronic supplementary material

Below is the link to the electronic supplementary material.

Supplementary Material 1

## Data Availability

The data and materials in this paper are available from the corresponding author on request.

## Abbreviations

HEUS: High-end ultrasound
HUD: Handheld ultrasound device
IQR: Interquartile range
POCUS: Point-of-Care-Ultrasound
US: Ultrasound
