# Supplementary material for: Phantom-based training of ultrasound-guided breast biopsy in medical education: a randomized controlled trial comparing handheld and high-end ultrasound
Source: BMC Med Educ. 2025 Apr 16;25:551. doi: 10.1186/s12909-025-07163-1 (PMC12004677; doi:10.1186/s12909-025-07163-1)
Supplement: Supplementary file 1 — Supplementary Material 1 [file 12909_2025_7163_MOESM1_ESM.pdf]

# Phantom-based training of ultrasound-guided breast biopsy in medical education

Student number: ______ Date:___________________

Ultrasound system: ☐ HUS ☐ HUD

---

**1) Personal impression: „I felt the ultrasound imaging system used was comfortable for me."**

absolutely 0 — not at all 5

**2) Personal impression: „I felt safe performing the biopsy while using this ultrasound imaging system.“ (orientation, needle firing & tissue asservation)**

absolutely 0 — not at all 5

**3) Self assessment: overall difficulty of task**

not difficult 0 — very difficult 5

---

## Assessment of mental workload:

**4)** Mental Demand — How mentally demanding was the task?

Very Low — Very High

**5)** Physical Demand — How physically demanding was the task?

Very Low — Very High

**6)** Temporal Demand — How hurried or rushed was the pace of the task?

Very Low — Very High

**7)** Performance — How successful were you in accomplishing what you were asked to do?

Perfect — Failure

**8)** Effort — How hard did you have to work to accomplish your level of performance?

Very Low — Very High

**9)** Frustration — How insecure, discouraged, irritated, stressed, and annoyed wereyou?

Very Low — Very High
